# P-2316. Comparing Whole Blood and Plasma Samples to Monitor EBV and CMV after transplantation

**DOI:** 10.1093/ofid/ofae631.2468

**Published:** 2025-01-29

**Authors:** Yuto Fukuda, Yuka Torii, Ken-ichi Iwada, Kazunori Haruta, Makoto Yamaguchi, Takako Suzuki, Atsushi Narita, Hideki Muramatsu, Yasuhiro Ogura, Yoshiyuki Takahashi, Yoshinori Ito, Jun-ichi Kawada

**Affiliations:** Nagoya University Graduate School of Medicine, Nagoya, Aichi, Japan; Nagoya University Graduate School of Medicine, Nagoya, Aichi, Japan; Nagoya University Graduate School of Medicine, Nagoya, Aichi, Japan; Nagoya University Graduate School of Medicine, Nagoya, Aichi, Japan; Nagoya University Graduate School of Medicine, Nagoya, Aichi, Japan; Nagoya University Graduate School of Medicine, Nagoya, Aichi, Japan; Nagoya University Graduate School of Medicine, Nagoya, Aichi, Japan; Nagoya University Graduate School of Medicine, Nagoya, Aichi, Japan; Department of Transplantation Surgery, Nagoya University Hospital, Nagoya, Aichi, Japan; Department of Pediatrics, Nagoya University Graduate School of Medicine, Nagoya, Aichi, Japan; Aichi Medical University Graduate School of Medicine, Nagoya, Aichi, Japan; Nagoya University Graduate School of Medicine, Nagoya, Aichi, Japan

## Abstract

**Background:**

Viral reactivation may occur after hematopoietic stem cell transplantation (HSCT) or solid organ transplantation owing to immunosuppression and can lead to severe consequences. Routine weekly monitoring using quantitative PCR is recommended for cytomegalovirus (CMV) after both allogeneic HSCT and solid organ transplantation and for Epstein–Barr virus (EBV) after allogeneic HSCT. However, the sample type most suitable for monitoring remains unclear.

**Methods:**

This retrospective analysis included 54 patients who underwent weekly viral monitoring using whole blood samples after HSCT or liver transplantation at the Nagoya University Hospital, Japan from January to December 2021. Whole blood and plasma samples were compared for their respective EBV and CMV DNA loads in the weekly serial samples from patients who tested positive for EBV or CMV at least once during the weekly virus monitoring. Subsequently, the dynamics of viral DNA were analyzed between the two sample types.

**Results:**

Based on whole blood samples, EBV DNA was detected in 22 patients, whereas CMV DNA was detected in 24 patients. Both EBV and CMV were detected in 13 patients. A total of 164 and 183 pairs of whole blood and plasma samples, respectively, were compared for EBV and CMV DNA. EBV DNA was detected more frequently in whole blood (55%) than in plasma (18%). The detection rate of CMV DNA was similar between the two sample types. The correlations between the viral loads in the two sample types were 0.515 and 0.688 for EBV and CMV, respectively. Among paired samples in which EBV DNA was detected in whole blood, the plasma EBV detection rate was significantly higher in patients who underwent hematopoietic stem cell transplantation than in those who underwent liver transplantation (42% vs. 19%, p = 0.022). After the week with the highest viral load in whole blood, subsequent trends in viral load exhibited similar patterns in both EBV and CMV DNA levels in both whole blood and plasma samples.

**Conclusion:**

The EBV detection rate was higher in whole blood, and a high correlation was observed between CMV DNA loads in whole blood and plasma samples. These results indicate that whole blood is useful for monitoring both EBV and CMV, whereas plasma is a potential alternative sample for monitoring CMV.

**Disclosures:**

All Authors: No reported disclosures

